# Molecular Cloning and Expression Analysis of P-Selectin from Zebrafish (*Danio rerio*)

**DOI:** 10.3390/ijms11114618

**Published:** 2010-11-17

**Authors:** Guijin Sun, Jie Pan, Kechun Liu, Xue Wang, Sifeng Wang

**Affiliations:** 1 College of Life Sciences, Shandong Normal University, Jinan, Shandong 250014, China; 2 Biology Institute of Shandong Academy of Sciences, Jinan, Shandong 250014, China

**Keywords:** P-selectin, zebrafish, cloning, expression, adenosine diphosphate

## Abstract

The glycoprotein P-selectin belongs to the selectin family of cell adhesion molecules. In this study, we cloned the full-length cDNA of P-selectin from zebrafish (*Danio rerio*) by the method of rapid amplification of cDNA ends polymerase chain reaction (RACE-PCR). Zebrafish P-selectin cDNA is 2,800 bp and encodes a putative 868 amino acid protein with a theoretical molecular weight of 122.36 kDa and isoelectric point of 6.27. A signal peptide of 25 amino acids is predicted at the N-terminus of the putative protein. All structural domains involved in P-selectin function are conserved in the putative protein. The amino acid sequence of zebrafish P-selectin is 37% to 39% identical to that of mammalian P-selectins. Real-time quantitative PCR and whole-mount *in situ* hybridization analysis revealed that P-selectin was expressed in early embryonic development, the expression increased from 0.2 hpf (1-cell stage) to 72 hpf, and the expression significantly upregulated within 30 minutes of ADP induction. The results indicate that the structure of P-selectin protein is highly conserved among species and zebrafish P-selectin plays an important role in early embryonic development and probably has similar biological function to mammalian P-selectins.

## 1. Introduction

The glycoprotein P-selectin belongs to the selectin family of cell adhesion molecules. It is also called platelet activation-dependent granule-external membrane protein, granule membrane protein-140 or CD62P. P-selectin is a type 1 membrane-spanning protein that has five distinct structural domains involved in P-selectin function: an N-terminal lectin domain; an epidermal growth factor (EGF)-like domain; a complement repeat domain (CRP; also called CCP or SCR); a transmembrane domain; and a cytoplasmic tail. This structure is well conserved among selectin family members. P-selectin is normally located in the alpha granules of platelets and Weibel-Palade bodies of endothelial cells, with low or absent expression [1–3]. With activation of endothelial cells and platelets by a variety of stimuli, P-selectin moves to cell surfaces where the expression is markedly increased [1–3].

The major ligand for P-selectin is P-selectin glycoprotein ligand 1 (PSGL-1), which is expressed in leukocytes [4]. The interaction of P-selectin with PSGL-1 mediates the rolling of platelets and neutrophils on activated endothelial cells and interactions of activated platelets with neutrophils and monocytes [5–7]. P-selectin plays a significant role in the inflammatory response, blood coagulation and thrombosis [8–10]. P-selectin-deficient mice are defective in leukocyte rolling and hemostasis [11,12]. P-selectin is a pivotal component in cardiovascular disease, and interventions directed against P-selectin or its ligand may be a therapeutic target in cardiovascular disease [13,14].

Zebrafish (*Danio rerio*) has become a prominent vertebrate animal model for investigating human development and diseases [15], because of its advantages of high fecundity, external embryonic development, transparency of the embryos, advanced genomic resources, and similar organ systems and gene sequences to humans. Zebrafish is especially appropriate for the study of cardiovascular disease because zebrafish embryos and larvae are transparent, and cardiac function and blood circulation are readily observed after 24 hour post-fertilization (hpf) [16,17].

To our knowledge, zebrafish P-selectin has not been characterized. In the present study, we cloned the full-length cDNA of zebrafish P-selectin and analyzed its expression pattern. Further, we preliminarily explored its biological function by adenosine diphosphate (ADP) induction.

## 2. Results

### 2.1. Cloning of Zebrafish P-Selectin

With the mouse P-selectin protein sequence, we performed a BLAST search in the NCBI database for a homologous zebrafish sequence and obtained the predicted cDNA sequence. The primers were designed in accordance with the predicted sequence. We obtained a full-length cDNA sequence of zebrafish P-selectin by a combination of RT-PCR and RACE.

### 2.2. Nucleotide Sequence Analysis of Zebrafish P-Selectin

**Figure 1.**
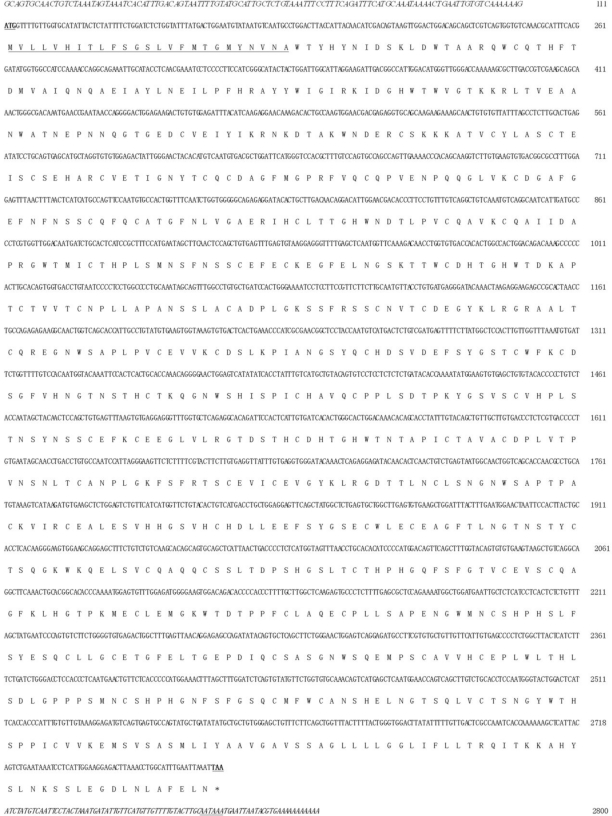
Full-length nucleotide and deduced amino acid sequences for zebrafish P-selectin. The putative signal peptide and polyadenylation signal are underlined, the start and stop codons are bold and underlined, and the 5’- and 3’-UTRs are in italics.

Zebrafish P-selectin cDNA is 2,800 bp and contains a 111-bp 5’-UTR, a 2,607-bp coding sequence and an 82-bp 3’-UTR (Figure 1). The sequence was deposited in GenBank (Accession No. GU566727). The zebrafish P-selectin gene contains 16 exons and 15 introns and is located on chromosome 20.

### 2.3. Amino Acid Sequence Analysis of Zebrafish P-Selectin

Zebrafish P-selectin cDNA encodes a putative 868 amino acid protein (Figure 1), with a theoretical molecular weight of 122.36 kDa and isoelectric point of 6.27. The putative protein is 38–39 amino acid longer than the human and horse protein [18,19]; 99–100 amino acids longer than the mouse [20], rat [21], and sheep protein (Accession No. NP_001009295); and 219–222 amino acids longer than bovine [22], rabbit (Accession No. AAA81385), and pig protein [23]. A signal peptide of 25 amino acids is predicted at the N-terminus of the putative protein. The putative protein is shown to be rich in cysteine residues (n = 69.8% of total amino acids) and to contain numerous (n = 19) potential asparagine-linked glycosylation sites (consensus sequence NXS/T), as observed in mammals.

**Figure 2.**
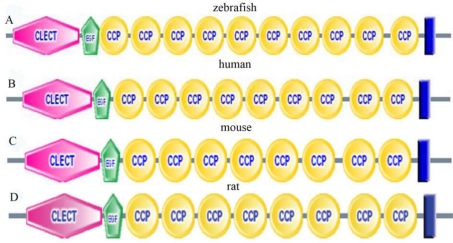
The conserved structural domains of zebrafish, human, mouse, and rat, P-selectins by Smart analysis. (**A**) zebrafish (**B**) human (**C**) mouse (**D**) rat.

The amino acid sequence of zebrafish P-selectin is 37–39% identical to those of mammalian P-selectins. Moreover, five structural domains putatively involved in P-selectin function are conserved in the putative zebrafish protein (Figure 2), including an N-terminal lectin domain, an EGF-like domain, a 10 complement repeat domain, a transmembrane domain and a cytoplasmic tail, which share 50–57%, 43–58%, 38–39%, 21–39% and 10–17% amino acids sequence homology with those of mammalian P-selectins. Among the five conserved structural domains, the length of the CCP domain is variable among species, mainly because of differences in the number of CCP. Zebrafish contains 10 CCPs; human and horse, nine; mouse, rat and ovine, eight; and bovine, pig and rabbit, six.

**Figure 3.**
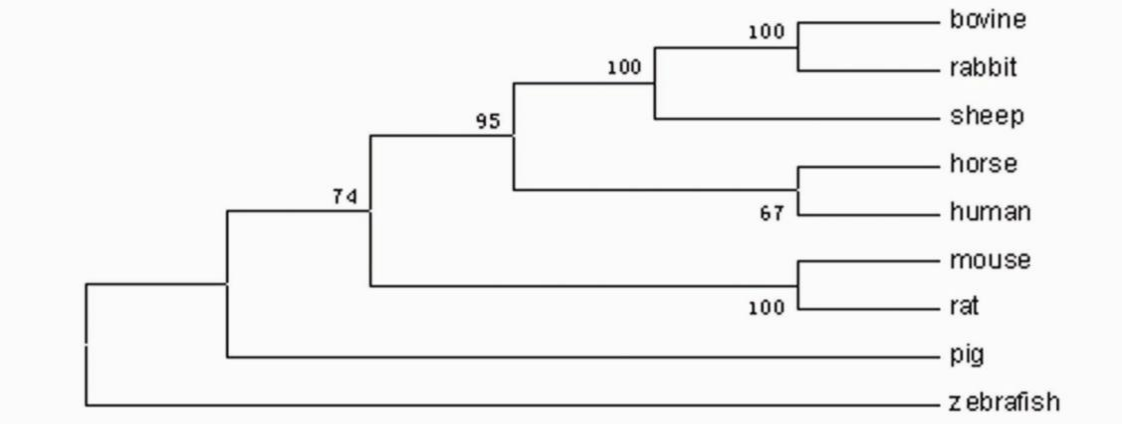
Phylogenetic tree of P-selectin family members. The numbers indicate the bootstrap confidence values obtained for each node after 1,000 replications showing: human (Accession No. NP_002996); mouse (Accession No. AAA40008); sheep (Accession No. NP_001009295); horse (Accession No. AAS79432); cattle (Accession No. NP_776608); rat (Accession No. NP_037246); rabbit (Accession No. AAA81385); and pig (Accession No. AAA79007).

In order to study the evolutionary relationship of zebrafish P-selectin with mammalian P-selectins, we constructed a phylogenetic tree by aligning the amino acid sequences of zebrafish P-selectin to that of mammalian P-selectins. As shown in Figure 3, the nine species were divided into two different branches, the first of mammalian species, and the other, zebrafish.

### 2.4. Expression Pattern of Zebrafish P-Selectin during Embryo Development

**Figure 4.**
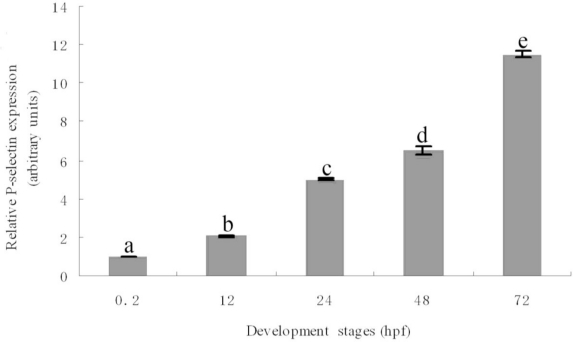
Expression analysis of zebrafish P-selectin during embryo development by real-time quantitative PCR. Different letters indicate significant differences (p < 0.05).

The temporal expression of P-selectin during zebrafish development was examined by real-time quantitative RT-PCR. The expression increased from 0.2 hpf (1-cell stage) to 72 hpf (Figure 4). The expression was approximately 10.5-fold more at 72 hpf than at 0.2 hpf and was significantly different from that at other developmental stages (Figure 4).

**Figure 5.**
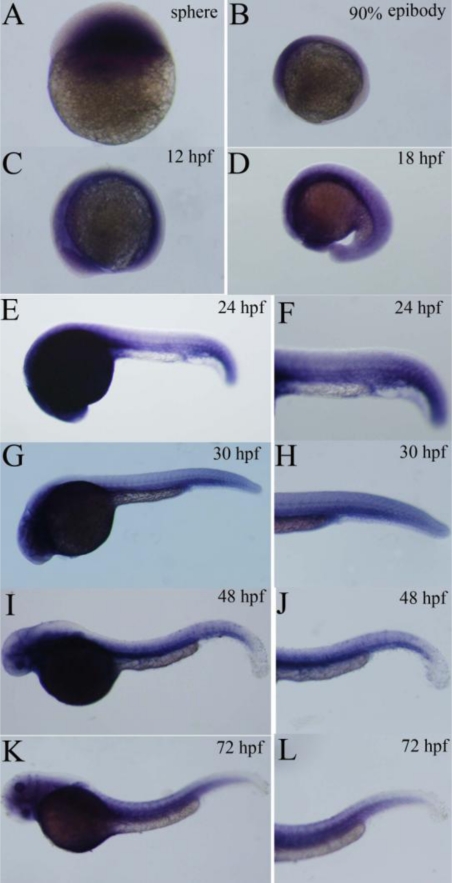
Expression analysis of zebrafish P-selectin during embryo development by whole mount *in situ* hybridization. Lateral view, anterior is to the left. (**A**) Sphere (**B**) 90% epibody (**C**) 12 hpf (**D**) 18 hpf (**E**, **F**) 24 hpf (**G**, **H**) 30 hpf (**I**, **J**) 48 hpf (**K**, **L**) 72 hpf (**F**, **H**, **J**, **L**) Enlarged view of trunk and caudal region.

The spatial and temporal expression patterns of P-selectin during zebrafish development was further examined by whole mount *in situ* hybridization. The results showed P-selectin transcript was detected at 4 hpf (Figure 5A). P-selectin was expressed in the anterior of the embryo at 90% epiboly (Figure 5B) and was ubiquitously expressed at 12 hpf (Figure 5C). At 18 hpf, P-selectin was strongly expressed in the head region and mainly expressed in dorsal aorta and axial veins in the trunk region (Figure 5D). At 24 hpf, the expression was restricted to the head region, anterior trunk region and caudal region, particularly the cardinal vein plexus region, and the signal was faint in the dorsal aorta and posterior cardinal vein (Figure 5E, F). At 30 hpf, the expression was increased in the dorsal aorta and posterior cardinal vein, with expression persisting in the head region and cardinal vein plexus region (Figure 5G, H). At 48 hpf, the expression persisted in the dorsal aorta, posterior cardinal vein, head region and cardinal vein plexus region (Figure 5I, J); the expression extended to the ventral region of trunk above the dorsal aorta, but the expression was faint in this region (Figure 5I, J). At 72 hpf, the expression was strong in the head region, ventral region of trunk and caudal region (Figure 5K, L), and the weak signal also appeared in the dorsal region of trunk (Figure 5K, L).

### 2.6. ADP-Induced Expression of P-Selectin

**Figure 6.**
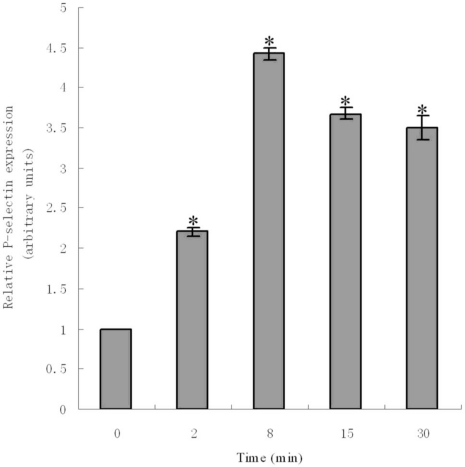
Real-time quantitative PCR results of P-selectin expression induced by adenosine diphosphate. *P < 0.05 compared with control (0 min).

To further investigate the biological function of zebrafish P-selectin, we analyzed the induction of its expression by ADP using real-time quantitative PCR. After ADP, the level of mRNA for P-selectin was detected. Results showed that levels of mRNA for P-selectin significantly increased within 30 min of ADP induction. The level of mRNA significantly increased 2 min after ADP induction, peaked at 8 min, and decreased at 15 and 30 min. The level of mRNA was approximately 3.4-fold higher than that of controls at 8 min (Figure 6).

## 3. Experimental Section

### 3.1. Cloning of zebrafish P-selectin cDNA

We performed a BLAST search of the NCBI database (http://www.ncbi.nlm.nih.gov) with the mouse P-selectin protein sequence (Genbank Accession No. AAA40008) and obtained the predicted zebrafish P-selectin cDNA sequence (Accession No. XM_001336788). According to the predicted sequence, the primers 5’-CCTGGACTTACCATTACAACATC-3’ and 5’-CAAGTACAAAACAACAT GAACA-3’ were designed to amplify zebrafish P-selectin cDNA by RT-PCR. RT-PCR involved use of total RNA extracted from embryos at 72 hpf, and amplification conditions were 3 min at 94 °C; 35 cycles of 94 °C for 30 s, 55 °C for 30 s, 72 °C for 3 min and a final extension at 72 °C for 10 min. On the basis of the cloned cDNA fragment sequence, the primers 5’-CGTTCGCGATGGGTTTCAGTG AGTCACA-3’ and 5’-CCTTTTGCTTGGCTCAAGAG-3’ for 5’-rapid amplification of cDNA ends (5’-RACE) and 3’-RACE were designed to amplify the 5’- and 3’-untranslated regions (UTRs) with use of the SMART-RACE cDNA Amplification Kit (Clontech). The primers 5’-CAGTGCAACTGTCTAAATAGTAAATC-3’ and 5’-ACGTATTAATTCATTTATTGCAAGTA-3’ were used in PCR to amplify the full-length cDNA of zebrafish P-selectin for verification. The amplification program was 94 °C for 5 min; 35 cycles of 94 °C for 30 s, 68 °C for 30 s, 72 °C for 3 min and a final extension at 72 °C for 10 min. All PCR products were cloned into pGEM-T easy vector (Promega) and sequenced.

### 3.2. Bioinformatics Analysis of Zebrafish P-Selectin

The nucleotide and amino acid sequences, exon–intron organization and chromosomal location, and conserved domain were analyzed online using the NCBI Blast server 2.0; the zebrafish whole-genome sequence project database (http://www.ensembl.org/Danio_rerio/blastview); and the Conserved Domains Database (http://smart.embl-heidelberg.de). The deduced signal peptide was identified by use of SignalP (http://genome.cbs.dtu.dk/services/SignalP/). Homology between the amino acid sequences of zebrafish P-selectin and other known P-selectins was analyzed by ClustalW (http://www.ebi.ac.uk/clustalw). The phylogenetic tree was constructed using Mega3 by the neighbor-joining method. The genetic distance was calculated by the ρ-distance method.

### 3.3. ADP Treatment

At 120 hpf, larvae were treated with 100 mM ADP for 2, 8, 15, and 30 min; the untreated group (0 min) was a control.

### 3.4. Real-Time Quantitative RT-PCR

Total RNA was extracted from embryos at 0.2 (1-cell), 12, 24, 48, 72 hpf and from larvae at 120 hpf, with and without ADP treatment, using TRIzol reagent (Invitrogen). Approximately 2 μg of total RNA was used for reverse transcription for first-strand cDNA with Moloney murine leukemia virus (Promega). Real-time quantitative PCR, with the first-strand cDNA used as a template, involved use of primers: for β-actin, (5’-TGGCTTCTGCTCTGTATGGC-3’ and 5’-CCCTGTTAGACAACTACCTCCCT-3’) (Accession No. NM_131031); for normalization and P-selectin (5’–TCGGGCATACTACTGGATTG-3’ and 5’-GGTTATTCGGTTCATTTGTCG-3’). The protocol was initial denaturation at 95 °C for 5 min, then 95 °C for 10 s, 59 °C for 15 s, 72 °C for 20 s, and 79 °C 5 s (fluorescent data were acquired), repeated for 40 cycles. For every assay, the negative control was without cDNA template. The 2^−^^ΔΔCt^ method was used to analyze data [35]. The expression at 0.2 hpf was used to calibrate temporal expression, and the expression for the control group was used to calibrate expression induced by ADP.

### 3.5. Whole-Mount *in Situ* Hybridization

Embryos were fixed in 4% paraformaldehyde (Sigma, U.K.). A 600 bp antisense RNA probe was generated from the zebrafish P-selectin coding sequence (679–1278 bp of the cDNA sequence, primers-5’-CAAGGTCTTGTGAAGTGTGAC-3’ and 5’-AAACTCATCGACAGAGTCAT-3’). The probe was synthesized by linearising the P-selectin-T easy plasmid with NdeI and transcribing with T7 RNA polymerase. Whole mount *in situ* hybridization was performed as described [36].

### 3.6. Statistical Analysis

Statistical analysis involved use of SPSS v12.0 (SPSS Inc., Chicago, IL). Data are presented as means ± SD. Comparisons between groups involved one-way ANOVA. The differences were considered statistically significant at p < 0.05.

## 4. Discussion

P-selectin cDNA was originally cloned from a human umbilical endothelial cell cDNA library [18]. Subsequently, the P-selectin gene was isolated and characterized in murine [20], bovine [22], rat [21], porcine [23], and equine models [19]. To further study the characteristic and expression of this gene, P-selectin was cloned from zebrafish, a widely studied animal model for human diseases. The cloned full-length cDNA sequence is 2800 bp. The cloned and predicted nucleotide sequences differ by 462 bp. We analyzed the predicted nucleotide and amino acid sequences. The results showed that the predicted nucleotide sequence was short of a 5’-UTR, and the amino acids from 1 to 95 at the predicted amino acid sequence did not belong to signal peptide nor to structural domains. Because the predicted sequence was obtained by analyzing zebrafish genome sequence using bioinformatics software, it cannot completely represent an authentic result. So it is possible that there are differences between the cloned and predicted nucleotide sequences. The cloned cDNA sequence stands for the authentic result.

Although the low amino acid sequence homology between zebrafish and mammals, five distinct structural domains involved in P-selectin function are conserved, indicating the biological function of zebrafish P-selectin is probably similar to that of mammalian P-selectins. This finding highlights that the primary structure of the P-selectin protein is relatively conserved across species and strongly suggests that the conserved structural domains are probably the basis of the consistent P-selectin function in all vertebrates. The potential N-linked glycosylation sites are conserved between zebrafish and mammals, further suggesting that carbohydrate modification may play an important role in P-selectin function. However, the positions of N-linked glycosylation sites are different between zebrafish and mammals. They are located on the extracellular portion in zebrafish and on the extracellular and cytoplasmic portions in mammals.

Alignments of P-selectins between zebrafish and mammals indicate the amino acid sequences of lectin and EGF-like domain are relatively highly conserved (50–57% and 43–58%); higher than the CCP, transmembrane and cytoplasmic domain sequences (38–39%, 21–39% and 10–17%). The relatively high conservation of the lectin and EGF-like domains suggests that these two regions are important for P-selectin function and also supports the concept that lectin and EGF domains of P-selectin play a critical role in ligand binding [24]. It immediately raises a significant question about the ligand of fish P-selectin. It is worth investigating if P-selectin has similar or identical ligands between fish and mammals. The function of the CCP domain remains undetermined. McEver demonstrated it was simply to act as a structural scaffold to facilitate the efficient interaction of the lectin and EGF-like domains with their counter-receptor [25]. Tedder *et al*. proposed functions include stabilization of receptor structure, mediating receptor oligomerization and extension of the lectin and EGF domains away from the membrane to optimize ligand binding [26]. The differences in the number of CCP between zebrafish and mammals support their concepts. The functions of five conserved domains in ligand binding and cell adhesion have been a major focus of study. Characterization of zebrafish P-selectin may provide insight into the understanding of the functions of these conserved domains.

Phylogenetic analysis indicated that mammalian and zebrafish P-selectins form distinct clades. These results indicated a relatively distant evolutionary relationship between mammals and zebrafish.

To clarify the role of P-selectin, we analyzed its spatial and temporal expression patterns during embryo development using real-time quantitative PCR and whole-mount *in situ* hybridization. The results showed that the expression increased gradually from the 1-cell stage to 72 hpf. The expression was evident in the anterior of the embryo at 90% epiboly and ubiquitous at 12 hpf. At 18 hpf, the expression was obvious in the head region and dorsal aorta and axial veins in the trunk region. At 24–48 hpf, the expression was strong in the circulatory system. At 72 hpf, the expression signal further extended to the dorsal region of trunk. Our results suggest that P-selectin is expressed maternally, plays important roles in early embryonic development, and may be involved in many aspects of early embryonic development, especially vascular development.

To our knowledge, the spatial and temporal expression patterns of P-selectin have never been reported in other species, so our examination of the expression pattern of zebrafish P-selectin provides some clues to the function of P-selectin in development. P-selectin is expressed in vascular endothelial cells and platelets in mammals [1–3]. However, the type of cell that contains zebrafish P-selectin expression is unknown. To further understand the precise role of P-selectin in early embryonic development and define the type of cell, further research is needed.

In general, P-selectin expression is absent or low on the surface of resting endothelial cells and blood platelets, but is obviously increased when the endothelium and blood platelets are stimulated by a variety of stimuli such as interleukin-3 [8], tumor necrosis factor-α (TNF-α) [27], endotoxin (lipopolysaccharides, LPS) [27], interleukin-4 [28], oncostatin M [28] and ADP [29]. Stimulation-dependent surface expression of P-selectin is a rapid event [8,27–29], and this mechanism remains largely elusive. Previous investigations have shown that ADP is an important agonist for platelet activation [30,31]; activated platelets play an important role in inflammation, hemostasis and thrombosis [32]. Since P-selectin is a component of the membrane of platelet alpha dense granules, the expression of this molecule at the platelet surface reflects activation of platelet [33,34].

In the present study, we studied the expression of mRNA for P-selectin after ADP induction. Results revealed the expression was rapid and significantly upregulated within 30 minutes of induction. This indicates that ADP is also an agonist of zebrafish P-selectin. Our study is primarily investigating the biological function of P-selectin in non-mammalian vertebrates. Our results and those of others suggest that zebrafish P-selectin is likely to play a key role in blood coagulation and thrombosis and may be *functionally* similar or identical to mammalian P-selectins. In previous studies, P-selectin protein synthesis was detected when endothelial cells and blood platelets were stimulated. In the present study, mRNA for P-selectin was detected. So, further investigation of protein expression after ADP induction is required.

## 5. Conclusions

We successfully cloned zebrafish P-selectin, which plays important roles during early embryonic development and likely has a similar biological function to mammalian P-selectins. Further, the cloning and characterization of zebrafish P-selectin is a prerequisite to generating transgenic zebrafish lines (with green fluorescent protein-tagged thrombocytes) to allow further the understanding of the pathogenesis of thrombosis and screening anti-thrombosis drugs. Our study may provide new ideas and information on potential drug therapy for the treatment of cardiovascular disease.
